# Unravelling the Turn‐On Fluorescence Mechanism of a Fluorescein‐Based Probe in GABA_A_ Receptors

**DOI:** 10.1002/ange.202205198

**Published:** 2022-05-13

**Authors:** Nadja K. Singer, Pedro A. Sánchez‐Murcia, Margot Ernst, Leticia González

**Affiliations:** ^1^ Institute of Theoretical Chemistry Faculty of Chemistry University of Vienna Währinger Str. 17 1090 Vienna Austria; ^2^ Vienna Doctoral School in Chemistry (DoSChem) University of Vienna Währinger Str. 42 1090 Vienna Austria; ^3^ Department of Molecular Neurosciences (Center for Brain Research) Medical University of Vienna Spitalgasse 4 1090 Vienna Austria; ^4^ Vienna Research Platform on Accelerating Photoreaction Discovery University of Vienna Währinger Str. 17 1090 Vienna Austria; ^5^ Present address: Division of Physiological Chemistry Otto-Loewi Research Center Medical University of Graz Neue Stiftingtalstr. 6/III 8010 Graz Austria

**Keywords:** Charge Transfer States, Fluorescent Probe, GABA-a Receptor, Gabazine, QM/MM

## Abstract

GABA_A_ (γ‐aminobutyric acid type A) receptors are ligand‐gated ion channels mediating fast inhibitory transmission in the mammalian brain. Here we report the molecular and electronic mechanism governing the turn‐on emission of a fluorescein‐based imaging probe able to target the human GABA_A_ receptor. Multiscale calculations evidence a drastic conformational change of the probe from folded in solution to extended upon binding to the receptor. Intramolecular ππ‐stacking interactions present in the folded probe are responsible for quenching fluorescence in solution. In contrast, unfolding within the GABA_A_ receptor changes the nature of the bright excited state triggering emission. Remarkably, this turn‐on effect only manifests for the dianionic prototropic form of the imaging probe, which is found to be the strongest binder to the GABA_A_ receptor. This study is expected to assist the design of new photoactivatable screening tools for allosteric modulators of the GABA_A_ receptor.

## Graphical abstract

A fluorescein‐based imaging probe turns‐on upon binding to the human γ‐aminobutyric acid type A (GABA_A_) receptor. Multiscale calculations show that fluorescence is due to the rod‐like conformation of the probe within the receptor. In contrast, intramolecular ππ‐stacking interactions in the folded conformation present in solution quench emission.

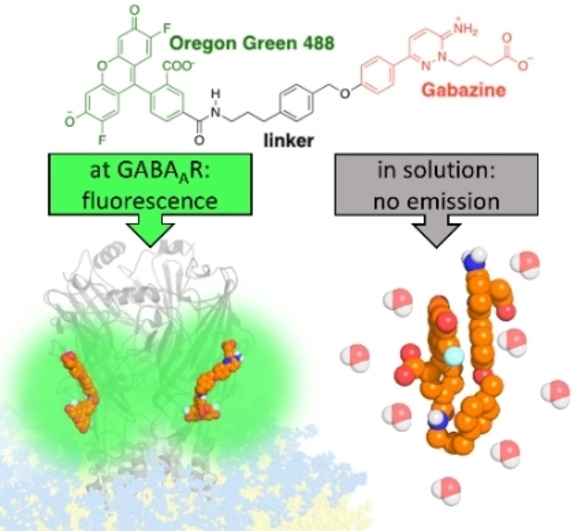

## Introduction

GABA_A_ receptors (GABA_A_Rs) are a family of pentameric transmembrane ligand‐gated ion channels based on the agonist γ‐aminobutyric acid (GABA), which is the major inhibitory neurotransmitter in the mammalian central nervous system.^[1]^ Involved in anxiety, epilepsy, autism, schizophrenia, and other neuropsychiatric disorders, GABA_A_Rs are important targets for pharmacological interrogation tools.[^2^, ^3^]

**Scheme 1:**
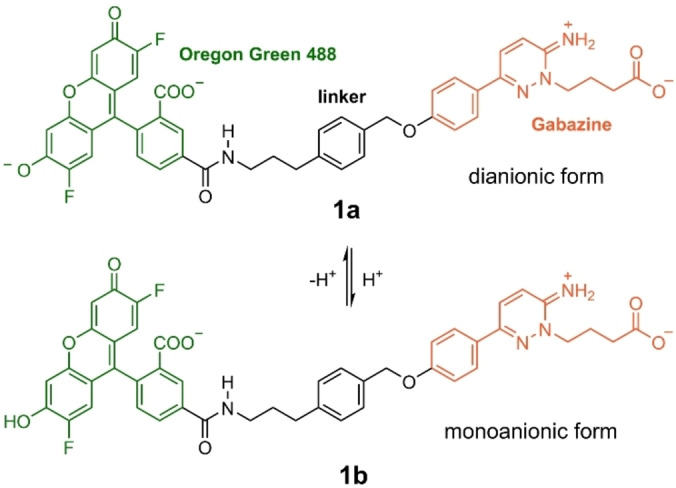
Chemical structure of the prototropic forms **1 a** and **1 b** of Gzn‐OG488, consisting of Oregon Green 488 (green) linked to gabazine (Gzn, orange), that could be present at physiological pH values (5.5–7.5).

Finding imaging tools that enable screening of positive allosteric modulators for GABA_A_Rs is particularly challenging. The quality of an imaging tool lies in the ability to change its photophysical properties upon binding, i.e., the probe must bind specifically to the target and its fluorescence should change substantially under biological conditions. One exciting new approach to this aim is to conjugate fluorophores to tags that specifically bind to the target in mind. This strategy was used by Sakamoto and collaborators,^[4]^ who recently reported the first turn‐on fluorescence imaging probe for a GABA_A_R.^[5]^ This probe consists of a 2′,7′‐difluorofluorescein fluorophore (Oregon Green 488, OG488)^[6]^ conjugated with the antagonist gabazine (Gzn) (Scheme 1) that inhibits the channel opening.

This novel probe shows no emission in solution but emits in the green region of the UV/Vis spectrum (*λ*_em_=488 nm) upon binding to a GABA_A_R. As stated in ref. [4], it is thus a powerful molecular tool in the high‐throughput screening of novel positive allosteric modulators of GABA_A_Rs. The described approach exploits the unique property of positive allosteric modulators to only enhance the affinity of primary (orthosteric) agonists but not antagonists to the receptor.[^4^, ^7^] Therefore, if a positive allosteric modulator binds to the GABA_A_R in the experimental setting, the binding of the agonist GABA is enhanced. Consequently, the antagonist‐based imaging probe will be released to the solution, with the resulting significant loss of fluorescence.^[4]^ Despite its great potential to target drug candidates, the rationale behind the turn‐on fluorescence of **1** upon binding to GABA_A_Rs is unknown.

**Figure 1:**
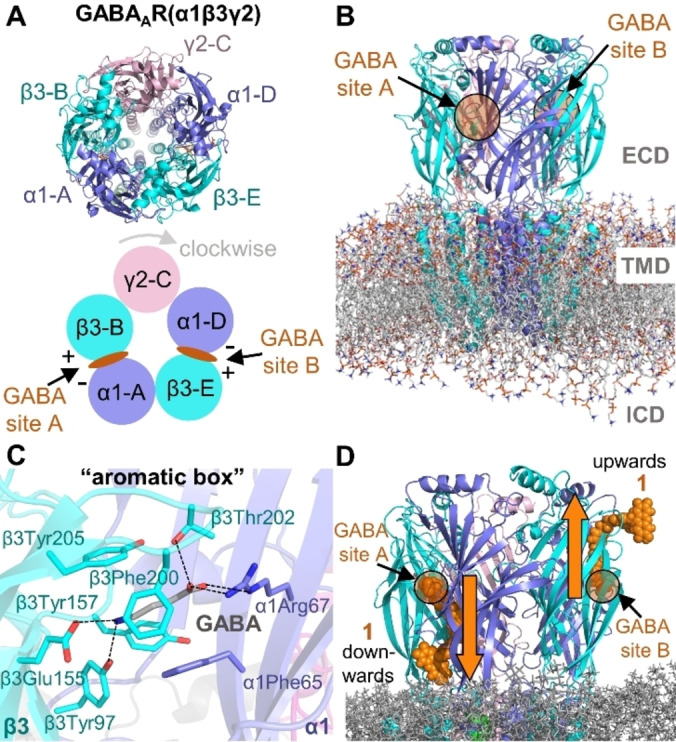
A) Top view of the heteropentameric GABA_A_R(α1/β3/γ2) with the naming scheme used for the subunits and the two GABA binding sites A and B. B) Structure of the receptor with marked extracellular domain (ECD) and transmembrane domain (TMD). The intracellular domain (ICD) is still unresolved. C) GABA (shown as sticks, C‐atoms in grey) binding mode found in the cryo‐EM structure 6HUO.^[15]^ D) Downwards and upwards orientations of **1** (shown as spheres) at the GABA binding sites A and B, respectively.

Computational studies are ideally suited to unveil the molecular basis of photopharmacological processes. However, there are several reasons why this is still a major endeavor. Fluorescence computations of few other‐purpose biological probes exist,[^8^, ^9^] but mechanistic studies of molecular probes bound to macromolecules are scarce.[^10^, ^11^, ^12^, ^13^, ^14^] In this system, one difficulty is that the subunit composition of GABA_A_Rs varies depending on their body localization, resulting in hundreds of possible structures. The most abundant and GABA‐active composition in the human adult brain is the heteropentameric γ2‐α1‐β2/3‐α1‐β2/3 clockwise arrangement of subunits (if viewed from the extracellular side), see Figure 1A.^[1]^ In this case, the binding of GABA occurs at the two β+/α‐ interfaces of the extracellular domain (ECD) (following the principal (+) and complementary (−) annotation of subunit faces)^[1]^ (sites A and B in Figure 1A). The structure of the intracellular domain, shown in Figure 1B, contrary to the ECD and transmembrane domain of such GABA_A_Rs, is still unresolved. Thus, to identify a reasonable conformational state of the transmembrane ligand‐gated ion channels when small molecules bind is challenging. Usually, the experimental conditions used for protein structure elucidation (i.e., protein crystallization protocols) differ from the physiological ones.

Particularly troublesome is that fluorescein‐based probes, such as the one here, are subject to acid‐base equilibria and can exist in different protonation states that might affect the absorption and emission properties of the fluorophore in solution.[^16^, ^17^] The fluorophore OG488 is marketed as insensitive to pH changes in the near‐neutral pH range but pH sensitive in moderately acidic solutions,^[18]^ thus displaying pH‐emission dependency. Indeed, the prototropic forms of OG488 have caused considerable controversy. The key study of Orte and co‐workers^[17]^ determined the ground state p*K*_a_ values of OG488 (1.02, 3.61 and 4.69), investigating its dianionic (as in **1 a** of Scheme 1), monoanionic (as in **1 b**), neutral, and cationic forms as well as excited‐state proton‐transfer reactions between them using time‐resolved fluorescence spectroscopy and global compartmental analysis. At physiological pH values (5.5–7.5), the unchanging absorption and fluorescence evidenced that the dianionic form is the predominant species with an experimental emission maximum at 515 nm in aqueous buffer.^[17]^ However, the monoanionic tautomer of the parental fluorescein compound has been calculated^[16]^ to have similar fluorescence wavelengths as the dianionic form. Additionally, the monoanion could be formed due to protonation in the environment, as happens with buried carboxylic acids in proteins.^[19]^ In passing we note that, the protonation of the carboxylic acid in fluorescein influences the relative rotation between its xanthene and benzoic acid fragments, and this in turn may also impact fluorescence.[^20^, ^21^]

Here, we use quantum mechanics/molecular mechanics (QM/MM) and molecular dynamics (MD) simulations to unravel the origin of the fluorescence of **1** when bound to a GABA_A_R. The MD simulations serve to find the binding mode of **1** to the GABA_A_R. The QM/MM calculations evaluate the excited state properties of the probe in different environments (for the QM benchmarking see Section S1). As we will show, two aspects are essential in order to disclose the turn‐on fluorescence effect: one refers to the conformational state of the fluorophore‐receptor complex, the other to the correct protonation state of the fluorophore. Accordingly, we consider here both the dianionic (**1 a**) and monoanionic (**1 b**) forms of OG488 (see Scheme 1), attached to the zwitterionic protonation state of Gzn, which we found the major one at pH 7.4 (Section S2, Figure S1). As we will show below, our results strongly support that the dianionic form **1 a** is the major binder to the GABA_A_R and thus responsible of the fluorescence. Further, in aqueous solution, the most stable conformation of **1 a** is folded with ππ‐stacking interactions between the fluorophore and Gzn, quenching fluorescence (Section S3, Figure S2). In contrast, upon binding to the receptor, **1 a** unclenches from the folded structure to a rod‐like structure. This drastic conformational change from folded to rod eliminates intramolecular ππ‐stacking interactions, thereby achieving the fluorescent turn‐on effect.

## Results and Discussion

### Structural Details of the GABA_A_R

To model the binding of **1** to its target, a precise knowledge of the GABA_A_R is required. Binding depends on the conformational state of the target; hence, a wrong conformation of the receptor will lead to erroneous conclusions. Several structures have been resolved so far for different compositions of heteropentamers[^15^, ^22^, ^23^] and the β3‐homopentamer^[24]^ of GABA_A_Rs. In particular, six experimental structures for the α1/β3/γ2 GABA_A_R have been released[^15^, ^23^] (Table S3). In the recent cryo‐EM structure of the heteropentameric GABA_A_R(α1/β3/γ2) (PDB id. 6HUO),^[15]^ two GABA molecules are found at the β3‐B^+^/α1‐A^−^ (site A, Figure 1A) and β3‐E^+^/α1‐D^−^ (site B) interfaces of the ECD of the receptor (following the A‐B‐C‐D‐E subunit notation of Masiulis et al.^[15]^). In this structure, GABA is surrounded by the “aromatic box” formed by β3Tyr157, β3Phe200, β3Tyr205, and α1Phe65 (Figure 1C). The iminium cation of GABA engages in a cation‐π interaction with the phenol ring of β3Tyr205 and in a salt bridge with the β3Glu155 carboxylate. On the other end, the carboxylate of GABA forms a salt bridge with the side chain of α1Arg67 as well as hydrogen‐bonds with α1Thr130 and β3Thr202 side chains.^[15]^ For our study we selected this structure of the α1/β3/γ2 GABA_A_R^[15]^ and removed the GABA molecules. Since Sakamoto et al.^[4]^ proved a competitive binding of **1** against GABA at sites A and/or B (the orthosteric binding sites), the antagonist Gzn should share some of the pharmacophoric points of GABA for the binding. Accordingly, we manually docked **1 a** and **1 b** keeping the GABA pharmacophoric interactions at sites A and B (Figure 1D). We discarded the use of an alternative structure of the same receptor in the presence of the antagonist bicuculline (PDB id. 6HUK) as no GABA molecules are present. Gzn shares less pharmacophoric points with bicuculline than with GABA. Additionally, we focus on uncovering the binding mode of the Gzn‐OG488 and not the overall functionality of the receptor.

The only possibility to place **1 a** and **1 b** in sites A and B was by “stretching” the probes and inserting them into the two β+/α‐ interfaces of the protein. In this way, upon binding of the Gzn moiety, **1** could be projected downwards or upwards with regards to the membrane (Figure 1D). MD simulations (data not shown) indicated that the upwards orientation ends up with the probe being in the bulk solvent. Thus, a downward orientation was defined for both **1 a** and **1 b**. Each prototropic form was run in a separate MD simulation in both sites. A simplified representation of a bilayer of 1‐palmitoyl‐2‐oleoyl‐sn‐glycero‐3‐phosphatidylcholine (POPC) lipids was chosen as membrane, as done in related studies.[^25^, ^26^, ^27^, ^28^] Care was taken to include the five disulfide bonds (one per subunit) present in the ECD of the receptor (Section S4). Force field parameters for the fluorescent probe were computed with the structures of **1 a** and **1 b** optimized at the B3LYP‐D3/def2‐SVP[^29^, ^30^, ^31^] level. AMBER atom types were used for **1 a** and **1 b** and their point charges (RESP) were computed at the same level of theory following the standard protocol in antechamber. The receptor was described via the AMBER ff14SB force field^[32]^ and the lipids via the Lipid17 force field.^[33]^ TIP3P water^[34]^ parameters and the ion parameters of Li/Merz were used.^[35]^ All the MD simulations were carried out using the AMBER18 software package.^[36]^

### MD Simulations of the Fluorescent Probe Bound to the GABA_A_R (α1/β3/γ2)

**Figure 2:**
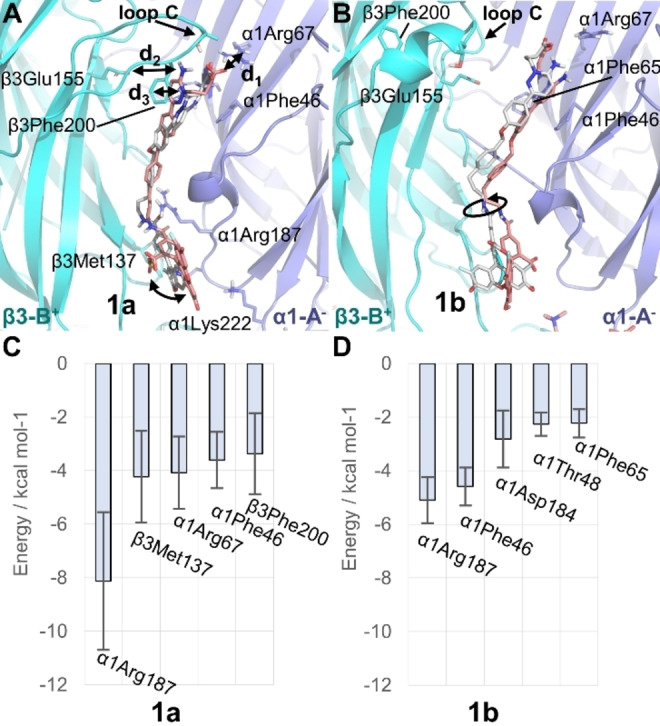
A), B) Representative binding mode of **1 a** (A) and **1 b** (B) at the GABA binding sites A (C‐atoms colored in pink) and B (C‐atoms colored in grey) of the GABA_A_R(α1/β3/γ2) pictured in site A. The main interacting residues and distances d_1_, d_2_, and d_3_ are highlighted as well as the range of motion of OG488 (black curved arrows). C), D) Binding energies (kcal mol^−1^) of **1 a** (C) and **1 b** (D) per residue of the most important interactions in site A.

Each 100 ns MD simulation (Scheme S1, Section S5) of **1 a** and **1 b** was carried out with the fluorescent part of the probe pointing downwards at the β3‐B^+^/α1‐A^−^ interface (site A) as well as at the β3‐E^+^/α1‐D^−^ interface (site B) of the GABA_A_R. When bound to the receptor, the probe adopts an extended, rod‐like conformation (Figure 2A and B). The Gzn part of **1 a** and **1 b** partially retains the pharmacophoric points shown by GABA at its binding site (Figure 1C). As an indication, we monitored two distances: the salt bridge between Gzn's carboxylate with the side chain of α1Arg67 (d_1_, Figure 2A) and the interaction of the probe's iminium cation with the carboxylate of β3Glu155 (d_2_). In site A, whereas the salt bridge along d_1_ is present ca. 70 % of the simulation time in both simulated systems (GABA_A_R with **1 a** and **1 b**), d_2_ is too large to ensure a salt bridge for any of the prototropic species (Figure S3). Thus, **1** may preserve only the interaction with α1Arg67, as found for the antagonist bicuculline.^[15]^ A similar trend is observed for d_1_ and d_2_ in site B. Interestingly, we perceived that in the presence of **1 b**, the loop C of the β3 subunit moves apart from α1Arg67 (Figure 2B). Noteworthy, the residue β3Phe200 is located on the loop C of the β3 subunit and is involved in the “aromatic box” upon GABA binding. Thus, we additionally measured the distance between the center of the pyridazinium ring in **1 a**/**1 b** and the center of the β3Phe200 phenyl ring (d_3_ in Figure S3) and found that in **1 a** d_3_ oscillates around 5 Å. Importantly, we measured that in **1 a** there is a stable sandwich ππ‐stacking interaction between the pyridazinium ring of the probe and the β3Phe200 side chain in both sites. In contrast, this interaction is present only 50 % of the simulation time in **1 b**. Additionally, we found that the “aromatic box” of **1 a** in the GABA binding site A (see Figures 1C and S4) adopts a structure closer to the receptor in presence of the antagonist bicuculline (PDB id. 6HUK) than to the initial cryo‐EM structure of the receptor (PDB id. 6HUO).^[15]^ This supports the validity of our model and its ability to adapt to the fluorescent probe.

Moreover, the inspection of the MD simulations reveals how the fluorophore in **1 b** at site B rotates with respect to the amide bond of the linker in comparison to **1 a** in the same binding site B (Figure S5). The root‐mean squared deviation (RMSD, Å) evolution at both binding sites along the MD simulation (Figure S6) shows a similar behavior for both prototropic forms, except for **1 b** at site B. Compared to **1 a**, **1 b** populates a second conformation cluster after 50 ns of simulation time (Figures 2B and S5). The subtle differences found for the binding at site A and B (for **1 a** and **1 b**) may have to do with the different environment. That is, the γ2 subunit in site B is right adjacent the α1 subunit (Figure 1A), while in site A γ2 is adjacent to the β3 subunit. This impacts the β3^+^/α1^−^ interface and therefore also the probe's conformation. Previous experimental studies confirmed that sites A and B have divergent affinities for multiple molecules (GABA, bicuculline, and muscimol).^[37]^ Additionally, the selected cryo‐EM structure for the simulation had alprazolam as positive allosteric modulator bound to site A, possibly resulting in some residual effects.

Also interesting is that the OG488 moiety adopts different orientations in **1 a** and **1 b** (Figures 2A and B). The fluorophore occupies a pocket defined by the three loops of Ser51‐Met55, Thr271‐Ile281 and Cys136‐Glu147 located on the β3 subunit, and by the loop Val181‐Tyr191 on the α1 subunit. The extra negatively charged oxygen atom present on the OG488 xanthene in **1 a** compared to **1 b** (Scheme 1) introduces additional interactions with the amide nitrogen atoms of residues Thr271‐Ile281 on the β3 unit. Consequently, the fluorophore has reduced mobility. In contrast, when the oxygen is protonated as in **1 b**, these interactions are replaced by punctual hydrogen bonding with the side chain of α1Asp184. This introduces a larger mobility on the MD simulation of **1 b** (Figure S5).

To find out whether **1 a** or **1 b** binds stronger to the GABA_A_R, we computed the binding energy of both prototropic forms to the receptor using the MM‐ISMSA method^[38]^ (Section S6, Tables S4 and S5). We found that the energy values for **1 a** are ≈2‐fold larger than for **1 b** in both sites (Table S4). While the additional negative charge of **1 a** compared to **1 b** is indeed responsible for a larger coulombic contribution to the binding energy, Table S4 underlines the stronger binding of **1 a** through increased van der Waals interactions (site A: −75.82 kcal mol^−1^ (**1 a**); −32.91 kcal mol^−1^ (**1 b**)). The decomposition of the binding energy per residue (Table S5) gives hints about the binding mode of the probe with the receptor, see Figures 2C and D for the main five residues. **1 a** and **1 b** strongly interact with α1Arg187 via a cation‐π stacking interaction with the phenyl ring of the linker. The binding energy analysis also confirmed the relevance of α1Arg67, β3Phe200, and α1Phe46 for the binding of the probe. Interestingly, we discovered a strong contribution of the hydrophobic β3Met137 for **1 a**. The latter residue interacts via its side chain with the fluorophore. Overall, the measurement of the distances together with the binding energy analysis clearly suggest that **1 a** binds stronger to the GABA site than **1 b**.

### MD Simulations of the Fluorescent Probe in Water Solution

**Figure 3:**
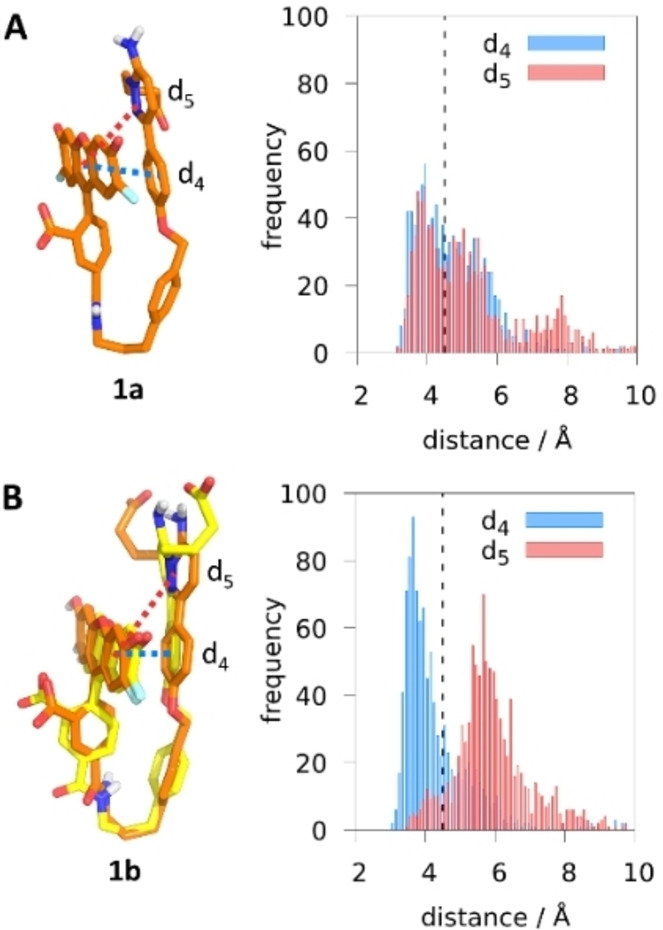
Representative structures of the major clusters of **1 a** (C‐atoms colored in orange, A) and **1 b** (C‐atoms colored in orange and yellow, B), respectively, along the 100‐ns MD simulations. Distance distributions [Å] measured from the center of mass of xanthene to the center of mass of phenyl (d_4_, blue) and of pyridazine (d_5_, red). The black dotted lines at 4.5 Å is used as a ππ ‐stacking interaction cutoff.

For the sake of comparison, we also simulated **1 a** and **1 b** in aqueous solution without the receptor. In contrast to the rod‐like conformation found in the presence of the GABA_A_R, the absense of the receptor induces the two OG488 and Gzn ends of **1 a** and **1 b** to bend together after very few ns. This folded arrangement is confirmed in Figure 3 by tracing the distances d_4_ and d_5_, defined from the center of mass of OG488’s xanthene to the center of masses of Gzn's phenyl ring and Gzn's pyridazine ring, respectively. The distances range from 3.5 Å to 8.0 Å along the simulation, confirming a ππ‐stacking interaction (cutoff 4.5 Å, black dotted line in Figure 3) between the heterocycles of Gzn and OG488 in both forms. However, there are differences. While **1 a** displays an oscillating ππ‐stacking of OG488 with both Gzn rings (Figure 3A), **1 b** only keeps the ππ‐stacking interaction from OG488 to the phenyl ring of Gzn (d_4_, Figure 3B). We hypothesize that the doubly negative charge of **1 a**, compared to the monoanionic **1 b**, can account for **1a**’s stronger drive to interact with the electron deficient pyridazine ring of Gzn. A clustering analysis of the MD simulation in water supports this hypothesis: while **1 a** presents one main cluster (58 %, Figure 3A), there are two in **1 b** (51 %: C‐atoms colored in orange, and 46 %: C‐atoms colored in yellow, Figure 3B). Because the pyridazine ring in **1 b** does not interact with OG488, it can rotate freely, giving rise to two main clusters.

In summary, our MD simulations evidence a drastic conformational change of **1 a** and **1 b** from a rod‐like conformation in the GABA_A_R environment to a folded structure in aqueous solution. The environment severely determines the conformation of the probe and its associated intramolecular ππ‐stacking interactions, which then affect the emission properties of the probe, as it will be shown next.

### QM/MM‐MD Simulations of the Probe's Fluorescence

**Scheme 2:**
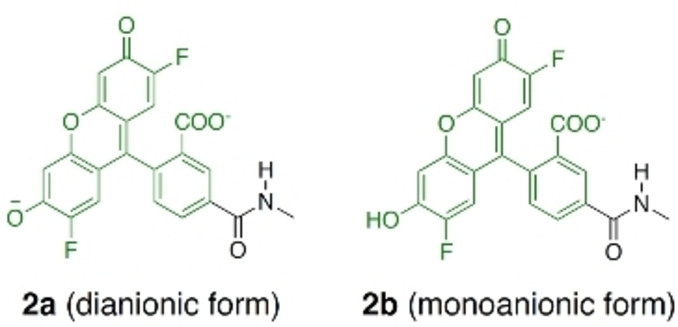
Chemical structures of the two model systems **2 a** and **2 b**.

To investigate the emission properties of **1 a** and **1 b** in both environments, we first used a model. We mimicked the context of the protein, where the fluorophore is not in contact with Gzn, by reducing the probe to only the OG488 part for each of the prototropic forms (labelled as **2 a** and **2 b** in Scheme 2). Comparing the results with those obtained with the real **1 a** and **1 b**, allows us to assess the impact of the intramolecular interactions of OG488 with Gzn on emission. Accordingly, we optimized the lowest singlet excited state (S_1_) of the full **1 a**, **1 b**, and truncated **2 a** and **2 b** species using time‐dependent DFT (TD‐DFT) and calculated each S_1_ emission wavelength (Table 1). TD‐DFT has been shown to be capable of computing emission properties in fluorescein‐based probes.[^16^, ^39^] We choose B3LYP‐D3 due to its reasonable agreement against our method of reference ADC(2) (Sections S1 and S3).

**Table 1:** Calculated emission energies (Δ*E*_em_, in nm and eV), oscillator strengths (arb. u.), and characters of transition for **1 a**, **1 b**, **2 a**, and **2 b**, using TD‐DFT and implicit solvation (water).[^29^, ^30^, ^31^, ^42^, ^43^, ^44^, ^45^, ^46^]

System		Δ*E*_em_		Oscillator strength	Character of transition^[c]^
		*λ* [nm]	[eV]	*f*_osc_ *[arb.u.]*	
					
dianionic	**1a** ^[a]^	810	1.53	0.013	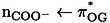
					
	**2a** ^[b]^	498	2.49	0.451	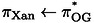
					
monoanionic	**1b** ^[a]^	1055	1.17	0.001	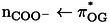
					
	**2b** ^[b]^	841	1.47	0.010	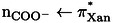

[a] B3LYP‐D3/def2‐SVP. [b] B3LYP‐D3/def2‐TZVP. [c] On basis of NTO analysis. Xan: xanthene of OG488, OG: OG488, COO^−^: carboxylate of OG488.

We use the oscillator strength (*f*_osc_) as an approximation for the emission intensity.[^10^, ^40^, ^41^] As can be seen from Table 1, the dianionic form **2 a** emits fluorescence at 498 nm with an oscillator strength *f*_osc_=0.451, while **1 a**, **1 b**, and **2 b** emit at lower energies (>800 nm) with decreased *f_osc_* <0.020. These calculations demonstrate that the intramolecular folding in **1 a** and **1 b** is responsible for the quenching of the fluorescence. We also found that the monoanionic fluorophore **2 b**, in the absence of the Gzn moiety, does not contribute to the fluorescence emission spectrum of OG488 in water. These findings contradict the idea that the monoanionic fluorophore could play a role in the fluorescent turn‐on effect. Thus, in the following we shall focus only on the dianionic form of the probe.

**Figure 4:**
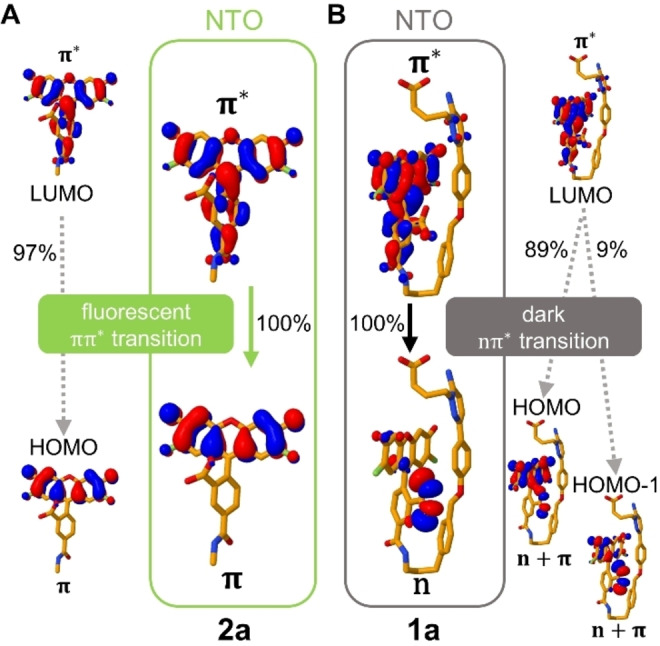
Natural transition orbitals (NTOs) and contributing molecular orbitals, involved in the S_1_→S_0_ emission in **2 a** (A, representing the rod‐like conformation found in the GABA_A_R environment) and in **1 a** (B, the folded conformation found in water). The calculations are done on the optimized S_1_ geometry at the TD‐B3LYP‐D3/def2‐TZVP(**2 a**)/def2‐SVP(**1 a**) level of theory including implicit water solvation.

To investigate the nature of the transitions involved in the S_1_→S_0_ emission, we analyzed the excited electron pairs of the natural transition orbitals (NTOs), see Figure 4. In both **1 a** and **2 a**, the origin NTOs of the excited electron are mostly composed of the respective LUMOs, localized on the xanthene and phenyl rings of the fluorophore (π character). Additionally, the NTO of **1 a** has a small contribution of Gzn, which may imply an energy transfer from Gzn to OG488. Nevertheless, the main differences are found in the final NTOs of the excited electron. In the absence of Gzn (**2 a**) the excited electron relaxes from a bright π→π* transition to an NTO located in the xanthene moiety (π character, mostly HOMO). On the contrary, in **1 a** the excited electron transitions to an NTO with a strong n‐character composed of both HOMO and HOMO‐1. In this NTO, most of the electron density is shifted into the non‐bonding n‐orbitals of the OG488 carboxylate. This implies a transition from a state with n→π* character in **1 a**, which is generally weak in emission.

After corroborating the intramolecular interactions of Gzn and the fluorophore OG488 in **1 a** as the source of the fluorescence turn‐off effect, we evaluated the explicit effect of the protein and water environments on the emission. To this aim, we carried out QM/MM‐MD simulations of **1 a** embedded in the solvated protein (Section S7). We used one snapshot from each the previous classical MD trajectories in water and bound to the protein and propagated **1 a** for 20 ps in the S_0_ electronic ground state using DFTB3/MM.[^47^, ^48^, ^49^, ^50^] This way, we removed any bias of the force field parameters on the geometry of the probe. Subsequently, we further propagated eleven snapshots of the last 10 ps of the DFTB3/MM simulation for each system on the S_1_ electronic state energy surface for 500 fs each using TD‐BP86‐D3/def2‐SVP/MM[^30^, ^51^, ^52^, ^53^] (see Table S6). Finally, five snapshots from the last 250 fs of each of the eleven simulation on the S_1_ surface were taken to compute the emission spectra using TD‐B3LYP‐D3/def2‐SVP@MM[^29^, ^30^, ^31^, ^46^] (55 S_1_ excited state calculations in total, see Section S7).

**Figure 5:**
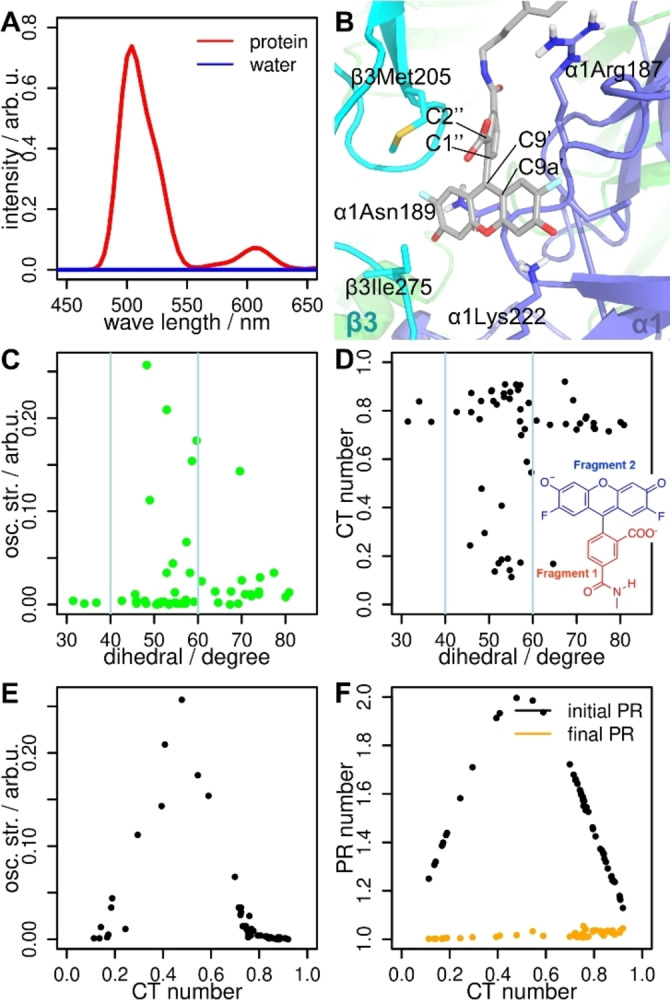
A) Computed emission spectra of **1 a** bound to the GABA_A_R (red line) and in water solution (blue line). B) Detail of the fluorophore of **1 a** in the protein environment. The residues with a significant interaction with the fluorophore are highlighted. The dihedral angle χ defined by atoms C2′′−C1′′−C9′−C9a′ is shown. C) Correlation between *f*_osc_ and the dihedral angle χ. D) Correlation between CT number and the dihedral angle χ. The fragments 1 and 2 relevant for the CT analysis are shown. E) Correlation between *f*_osc_ and the CT number. F) Correlation between the participation ratio (PR) for the initial/final electron delocalization and the CT number.

Figure 5A shows the simulated emission spectra of **1 a** in water (blue line) and bound to the GABA_A_R (red line). Our computed emission maximum inside the protein is 500 nm and reproduces nicely the experimental value (525 nm).^[4]^ In addition, this emission maximum is similar to the one calculated for the fluorophore in implicit solvation (**2 a**, 498 nm, Table 1). As observed experimentally,^[4]^ the emission of **1 a** in water is negligible.

Finally, we analyzed the relationship between the *f*_osc_ and the charge transfer (CT) character of the deexcitations with respect to the relative orientation of the two rings that compose the fluorophore in **1 a**. CT=0 means that there is no charge transfer between fragments, whereas CT=1 implies that one electron is transferred between fragments 1 (xanthene) and 2 (benzoic acid). The relative orientation of the two fluorophore rings is measured with the dihedral angle χ around the bond C1′′−C9′, as defined in Figure 5B. If the two rings are perpendicular, χ=90° and if they are coplanar, χ→0°. Within the protein environment we observe neither an ideal perpendicular nor coplanar conformation. There is no clear correlation between *f*_osc_ and the dihedral angle. Nevertheless, the brightest transitions appear at intermediate conformations between the perpendicular and coplanar orientations (40°<χ<60°, Figure 5C). Still, χ has a strong impact on the CT number (Figure 5D). Perpendicular‐like orientations (χ>60°) and coplanar‐like conformations (χ<40°) show high CT numbers (>0.7) while intermediate conformations (40°<χ<60°) show a drastic change of the CT character (<0.2) indicating emissive transitions. Interestingly, we also see that high as well as low CT numbers correlate to almost dark (*f*_osc_<0.1) transitions (Figure 5E) and local excitations (initial/final participation ratio (PR)→1, Figure 5F). A local excitation means that the initial and final electron will localize on only one of the fragments. In contrast, bright (*f*_osc_>0.1) conformations have CT numbers between 0.3 and 0.7 and delocalized initial orbitals (initial PR→2). Initial PR values of 2 indicate that the initial orbital is distributed over two molecule fragments and it is thus not a biradical. Figure 5F therefore confirms the conclusions drawn from the NTO picture (Figure 4A), i.e. that the electron which is deexcited comes from an orbital distributed over the two fragments (initial PR=2) and goes to only the xanthene part of the fluorophore (final PR=1), thereby emitting fluorescence.

## Conclusion

We have unraveled the structural and electronic features that explain the turn‐on fluorescence of **1** when going from solution to the heteropentameric GABA_A_R(α1/β3/γ2) environment. We have proposed a binding mode for the monoanionic and dianionic forms of the fluorescent probe **1** to the receptor based on the pharmacophoric binding points of the natural ligand GABA and on MD simulation results. The probe adopts a rod‐like conformation inside the ECD of the protein, with Gzn occupying the GABA site and OG488 pointing down to the lipid membrane. In contrast, the probe adopts a folded conformation in aqueous solution. Importantly, of the two prototropic forms of the probe, our MD simulation data support that the dianionic form binds stronger to the GABA_A_R than the monoanionic form. For the dianionic form of **1**, our computed emission spectra reproduce the turn‐on effect upon binding to the GABA_A_R with fluorescence at ≈500 nm. Quantum chemical calculations together with wavefunction analysis rationalize the experimental loss of the fluorescence: the emissive S_1_→S_0_ transition (π*→π character) of **1** at the receptor is replaced by a negligibly emissive π*→n transition in solution due to intramolecular ππ‐stacking interactions. Altogether, our work shows the strength of multiscale simulations to provide the first robust all‐atom model of the Gzn‐based fluorescence probe bound to the GABA_A_R(α1/β3/γ2), revealing the origin of its turn‐on effect. Further, it helps setting the photophysical basis for future development of photoactivatable compounds.

## Conflict of interest

The authors declare no conflict of interest.

1

## Supporting information

As a service to our authors and readers, this journal provides supporting information supplied by the authors. Such materials are peer reviewed and may be re‐organized for online delivery, but are not copy‐edited or typeset. Technical support issues arising from supporting information (other than missing files) should be addressed to the authors.

Supporting Information

## Data Availability

The data that support the findings of this study are available in the Supporting Information of this article.
