# Supplementary material for: Unravelling the Turn‐On Fluorescence Mechanism of a Fluorescein‐Based Probe in GABAA Receptors
Source: Angew Chem Weinheim Bergstr Ger. 2022 May 13;134(30):e202205198. doi: 10.1002/ange.202205198 (PMC10962554; doi:10.1002/ange.202205198)
Supplement: Supplementary file 1 — Supporting Information [file ANGE-134-0-s001.pdf]

Angewandte Chemie
Eine Zeitschrift der Gesellschaft Deutscher Chemiker

Supporting Information

Unravelling the Turn-On Fluorescence Mechanism of a Fluorescein-Based Probe in $GABA_A$ Receptors

N. K. Singer, P. A. Sánchez-Murcia, M. Ernst, L. González**

SUPPORTING INFORMATION

Table of contents

**Section S1**	Selection of the density functional<br>Tables S1, S2	2
**Section S2**	Ground state of the fluorescent probe **1**<br>Figure S1	5
**Section S3**	Emission of the fluorescent probe **1**<br>Figure S2	6
**Section S4**	Setting up of the systems<br>Table S3	7
**Section S5**	MD simulations<br>Figures S3, S4, S5, S6	8
**Section S6**	Binding energy analysis<br>Tables S4, S5	11
**Section S7**	QM/MM MD simulations<br>Scheme S1, Figure S7	13
**References**		16

Section S1 SELECTION OF THE DENSITY FUNCTIONAL

Here we present a benchmark of several density functionals to describe the absorption and emission of **1** against ADC(2). To this aim, we used only the two fluorophores of interest, **2a** and **2b**.

Absorption. Five density functionals were selected: two hybrid functionals (PBE0, B3LYP),[1,2] two meta-hybrid functionals (M06, M062X),[3] and one long-range corrected hybrid functional (CAM-B3LYP).[4] In all cases, the ground state (S_0) geometry for both fluorophores **2a** and **2b** was optimized using DFT within the Gaussian 16, v. A.01 suite,[5] the def2-TZVP[6] basis set, the Grimme D3 dispersion correction[7], and implicit water solvation using the default and the corrected Linear Response - Polarizable Continuum Model (LR-PCM, cLR-PCM).[8–11] Then, the molecules are vertically excited from the optimized S_0 geometry to the first excited state (S_1) using time-dependent density functional theory (TD-DFT). These results were compared to those obtained on the B3LYP-optimized geometry with ADC(2)/def2-TZVP (our reference method) with implicit water solvation (COSMO), using TURBOMOLE v7.0.[12] Regarding the comparability of PCM and COSMO implicit solvation models: We did not change the dielectric scaling factor (x = 0) used by Gaussian 16 for the integral equation formalism PCM method as it was shown to produce comparable results to the COSMO implicit solvation for ionic solutes (which we have with **2a** and **2b**).[13] Table S1 collects the energies, oscillator strengths and orbital character (natural transition orbitals, NTO) of the S_1 when using TD-DFT and ADC(2).

The five TD-DFT calculations coincide with the results of ADC(2), predicting the same character for the S_1 state ($\pi_{Xan} \rightarrow \pi^*_{Xan}$) for **2a** and **2b**. In all cases, we see that the S_1 state is the brightest state at the S_0 geometry. The absorption energies are all overestimated by TD-DFT with LR-PCM, with deviations from 0.49 eV (B3LYP) to 0.64 eV (CAM-B3LYP) in **2a**, and from 0.26 eV (B3LYP) to 0.52 eV (CAM-B3LYP) in **2b**. The cLR-PCM method overestimates the absorption energies even further. Nevertheless, the trend for all the different functionals is consistent. From these results, we considered the two DFT functionals with the smallest deviations from the ADC(2) results, B3LYP and M06, as most suitable candidates to evaluate the emission process.

Table S1. Calculated S_1 absorption energies (E_{ab} in eV and nm), oscillator strengths (f_{osc}, arb. u.), and state orbital characters of **2a** and **2b** using TD-DFT and ADC(2) in implicit water solvation.

Method	2a				2b			
	E_{ab} [nm]	E_{ab} [eV]	f_{osc} [arb.u.]	character of transition[4]	E_{ab} [nm]	E_{ab} [eV]	f_{osc} [arb.u.]	character of transition[4]
**LR-PCM**								
**CAM-B3LYP[1]**	419	2.96	0.865	$\pi_{Xan} \rightarrow \pi^*_{Xan}$	381	3.25	0.703	$\pi_{Xan} \rightarrow \pi^*_{Xan}$
**M062X[1]**	424	2.93	0.876	$\pi_{Xan} \rightarrow \pi^*_{Xan}$	382	3.24	0.705	$\pi_{Xan} \rightarrow \pi^*_{Xan}$
**PBE0[1]**	434	2.86	0.736	$\pi_{Xan} \rightarrow \pi^*_{Xan}$	403	3.07	0.538	$\pi_{Xan} \rightarrow \pi^*_{Xan}$
**M06[1]**	437	2.84	0.732	$\pi_{Xan} \rightarrow \pi^*_{Xan}$	406	3.06	0.559	$\pi_{Xan} \rightarrow \pi^*_{Xan}$
**B3LYP[1]**	442	2.81	0.682	$\pi_{Xan} \rightarrow \pi^*_{Xan}$	415	2.99	0.441	$\pi_{Xan} \rightarrow \pi^*_{Xan}$
**cLR-PCM**								
**CAM-B3LYP[2]**	403	3.08	-	-	371	3.34	-	-
**M062X[2]**	406	3.05	-	-	372	3.34	-	-
**PBE0[2]**	419	2.96	-	-	394	3.14	-	-
**M06[2]**	422	2.94	-	-	396	3.13	-	-
**B3LYP[2]**	428	2.90	-	-	409	3.03	-	-
**COSMO**								
**ADC(2)[3]**	535	2.32	0.715	$\pi_{Xan} \rightarrow \pi^*_{Xan}$	454	2.73	0.565	$\pi_{Xan} \rightarrow \pi^*_{Xan}$

[1]...-D3/def2-TZVP/LR-PCM(water)//B3LYP-D3/def2-TZVP/PCM(water);[2]...-D3/def2-TZVP/cLR-PCM(water)//B3LYP-D3/def2-TZVP/PCM(water); [3]ADC(2)/def2-TZVP/COSMO(water)//B3LYP-D3/def2-TZVP/PCM(water); [4]On basis of NTO analysis. Xan: xanthene of OG488

Emission. Emission energies and associated orbital characters are investigated with the best performing functionals for absorption, M06 and B3LYP,[1–3] as well as CAM-B3LYP,[4] benchmarked against ADC(2). We first optimized the minimum energy geometry on the S_1 PES of **2a** and **2b** with TD-DFT/def2-TZVP and LR-PCM. As for absorption, the emission energies were calculated with the default LR-PCM implicit solvation as well as with the corrected approach cLR-PCM for B3LYP and M06.

As it can be seen in Table S2, the TD-DFT calculations for **2a** is in line with the results of ADC(2), with transition character ($\pi_{Xan} \leftarrow \pi^*_{OG}$) and energies by +0.44 eV (similar to the shift seen with the absorption calculations). The errors for the non-emissive **2b** are less pronounced: ADC(2) gives a value of 760 nm (1.63 eV), again in reasonable agreement with B3LYP (841 nm, 1.47 eV). We stress that for **2b** we found a surprisingly low energy S_1 state with ADC(2) (1.63 eV). Out of

curiosity, we also calculated the emission with CAM-B3LYP for **2a** and **2b** with LR-PCM. While the results for **2a** are consistent with the other functionals and ADC(2) (besides the energy shift), the results for **2b** show a highly deviating emission energy of 423 nm (2.93 eV). A close inspection shows that CAM-B3LYP converges to a different state as our reference method (different character and thus different oscillator strength), which lead us to distrust the CAM-B3LYP results. Regarding the two PCM methods (LR or cLR-PCM), very similar results are obtained in both cases (M06, B3LYP) for **2a** ($\Delta E < 0.05$ eV). Again, we find an overestimation of the S_1 state energy with TD-DFT: +0.40 eV (B3LYP) and +0.55 eV (M06) in comparison with the ADC(2) emission energy.

Table S2. Calculated emission energies (E_{em} in eV and nm), oscillator strengths (f_{osc}, arb. u.), and transition characters of **2a** and **2b** using TD-DFT and ADC(2) with implicit water solvation.

Method	2a				2b			
	E_{em} [nm]	E_{em} [eV]	f_{osc} [arb.u.]	character of transition[4]	E_{em} [nm]	E_{em} [eV]	f_{osc} [arb.u.]	character of transition[4]
**LR-PCM**								
**M06[1]**	477	2.60	0.534	$\pi_{Xan} \leftarrow \pi^*_{OG}$	693	1.79	0.035	$n_{COO-} \leftarrow \pi^*_{Xan}$
**B3LYP[1]**	498	2.49	0.682	$\pi_{Xan} \leftarrow \pi^*_{OG}$	841	1.47	0.010	$n_{COO-} \leftarrow \pi^*_{Xan}$
**CAM-B3LYP[1]**	436	2.84	0.801	$\pi_{Xan} \leftarrow \pi^*_{OG}$	423	2.93	0.619	$\pi_{Xan} \leftarrow \pi^*_{OG}$
**cLR-PCM**								
**M06[2]**	478	2.60	-	-	1307	0.95	-	-
**B3LYP[2]**	507	2.45	-	-	3693	0.34	-	-
**COSMO**								
**ADC(2)[3]**	605	2.05	0.584	$\pi_{Xan} \leftarrow \pi^*_{OG}$	760	1.63	0.085	$n_{COO-} \leftarrow \pi^*_{Xan}$

[1]…-D3/def2-TZVP/LR-PCM(water); [2]…-D3/def2-TZVP/cLR-PCM(water); [3]ADC(2)/def2-TZVP/COSMO(water)//B3LYP-D3/def2-TZVP/PCM(water); [4]On basis of NTO analysis. **Xan**: xanthene of OG488; **OG**: OG488; **COO-**: carboxylate of OG488

Choice of the level of theory. According to the results obtained above, we consider that B3LYP is an adequate level of theory to perform single point TD-DFT calculations on the OG488 fluorophore, also within the $GABA_AR$. We used the default LR-PCM model, as we do not find appreciable changes when using the corrected LR approach. The results of the static calculations on **2a** and **2b** are presented in Table 1 of the main manuscript.

Section S2 GROUND STATE OF THE FLUORESCENT PROBE 1

The Cartesian coordinates (see repository: https://phaidra.univie.ac.at/o:1383031) of the fluorescent probe gabazine (Gzn) conjugated with Oregon Green (OG488)[14] (**1**) were generated as discussed in the Introduction of the main manuscript, with two of its more probable protonation states based on a pH value of 7.4 (see Figure S1 and Scheme 1) and taking the pharmacophoric points for the binding of GABA into account. The relevant protonatable moieties are the carboxylate of Gzn, the iminium cation of Gzn, the carboxylate of OG488, and the xanthene of OG488. The latter either has a hydroxy group in the monoanionic form (**1b**) or is fully deprotonated in the dianionic form (**1a**) (Scheme 1).

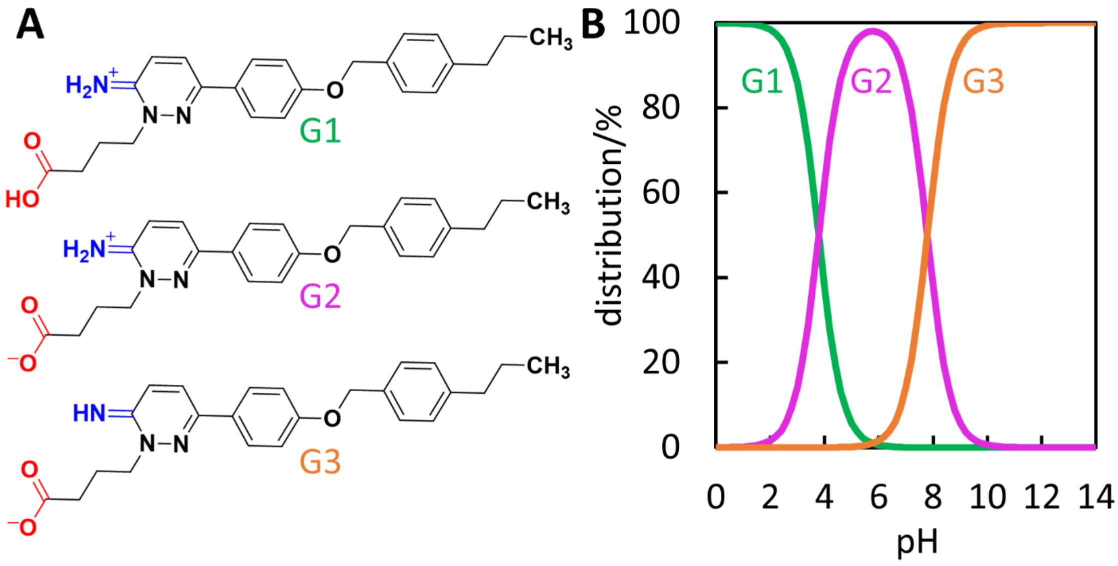

Figure S1. (A) Chemical structures of three protolytic forms (G1, G2, G3) of **1**'s gabazine (Gzn). The size of **1** was reduced to Gzn and parts of the linker for the pKa calculations. (2) Plot of the distribution of G1, G2, and G3 against the pH value calculated with ChemSketch.

The ground state geometry for both forms of **1** was optimized using DFT with the B3LYP[15] functional, the def2-SVP[6] basis set, the Grimme D3 dispersion correction[7] and PCM water solvation. The calculations were carried out in the Gaussian 16, v. A.01 suite.[5]

Section S3 EMISSION OF THE FLUORESCENT PROBE 1

The geometries of the first excited state S_1 of **1a** and **1b** were optimized using with TD-B3LYP-D3/def2-SVP/LR-PCM(water), which is the protocol described in Section S1, apart from the basis set size. The choice of the B3LYP functional is justified from the comparison with other DFT-functionals against ADC(2) as method of reference on **2a** and **2b**. We note that TD-DFT has been extensively used in the literature to describe the electronic structure of fluorescein-based[37,38] and organic photoprobes.[39,40] The results for the S_1 to S_0 emission for the whole fluorescent probes **1a** and **1b** can be seen in Table 1 in the main text. To further validate the DFT results also for **1**, we also calculated the emission of **1b** with ADC(2)/def2-SVP using the B3LYP-optimized geometry with implicit water solvation (COSMO), as implemented in TURBOMOLE v7.0.[12] We obtain an emission energy of 1.38 eV with an oscillator strength (f_{osc}) of 0.010, which is comparable to that obtained with B3LYP-D3 (Table 1) of 1.17 eV and f_{osc} of 0.001. Moreover, the natural transition orbitals (NTOs) for both methods have the same nπ* transition state character ($n_{COO^-} \leftarrow \pi^*_{OG}$) (shown in Figures 4 for **1a** and in S2 for **1b**). This result corroborates that the B3LYP functional is sufficiently accurate for the system under study.

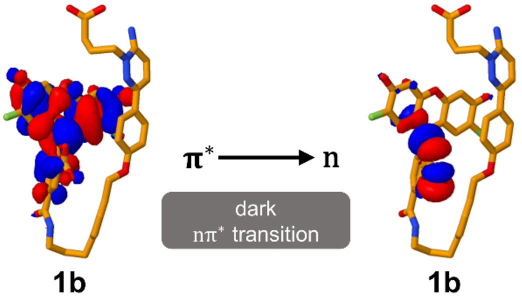

Figure S2. Natural transition orbitals (NTOs) involved in the $S_1 \rightarrow S_0$ emission of **1b** (the folded conformation found in water). The calculations are done on the optimized S_1 geometry at the ADC(2)/def2-SVP/COSMO(water)//TD-B3LYP-D3/def2-SVP(PCM(water) level of theory.

Section S4 SETTING UP OF THE SYSTEMS

The Cartesian coordinates of the receptor were obtained from the cryogenic electron microscopy (cryo-EM) solved structure of the human full-length heteromeric $GABA_AR$(α1/β3/γ2) in complex with the anxiolytic alprazolam (Xanax), the transmitter GABA, and the megabody Mb38 (PDB id. 6HUO).[16] This structure, among other reasons mentioned in the "Results and Discussion" section, was used in this work, because the structure of the same receptor in the presence of another antagonist (bicuculline, see Table S3, PDB id. 6HUK), like Gzn, presented a distorted orientation of α1Arg67 by one of the aromatics in bicuculline. Thus, to preserve the former residue as one of the pharmacophoric points for the binding of Gzn at the beginning of our simulation, we selected the structure in the presence of GABA. Alprazolam, GABA, and Mb38 were removed for our simulations. Since the secondary structure of the intracellular domain (ICD) of all subunits is unknown (α1, amino acids 313-391; β3, amino acids 308-423; γ2, amino acids 323-400) the peptide linker of sequence Ser-Gln-Pro-Ala-Arg-Ala-Ala used experimentally by Miller *et al*.[17] was introduced as replacement for each of the subunits ICD.[18,19] The protonation states of the titratable residues on the receptor were estimated at pH 7.4 using the H++ server.[20–23] Care was taken to include the five disulfide bonds (one per subunit) present in the extracellular domain (ECD) of the receptor. The $GABA_AR$ was embedded into an explicit 1-palmitoyl-2-oleoyl-sn-glycero-3-phosphatidylcholine (POPC) lipid membrane (190 lipids on the lower membrane, 200 in the upper membrane) after reorientation with the OPM server. [24] The system was solvated with TIP3P water[25] molecules with a box of size 130x125x195 Å using the server CHARMM-GUI.[26–30] In addition to 21 neutralizing Cl^- ions, a concentration of 0.15 mM of brine (210 Na^+ and Cl^- ions) was added to reproduce the experimental conditions of Sakamoto *et al*.[14] Before adding the probe, the unbound system was relaxed by means of standard short MD simulations in AMBER18.[31] The placing of the probe inside the GABA binding sites was done with the hypothesis of preserving the two pharmacophoric points of the binding of GABA: the salt-bridges between GABA and residues α1Arg67 and β3Glu155.

Table S3. Released α1/β3/γ2 $GABA_AR$ structures at time of simulation.[16,32]

PDB id.	Method / Resolution (Å)	Ligands and small molecules
6HUG	cyro-EM / 3.10	megabody 38, picrotoxin
6HUJ	cyro-EM / 3.04	megabody 38, picrotoxin, GABA
6HUK	cyro-EM / 3.69	megabody 38, bicuculline
6HUO	cyro-EM / 3.26	megabody 38, alprazolam, GABA
6HUP	cyro-EM / 3.58	megabody 38, diazepam, GABA
6I53	cyro-EM / 3.20	megabody 38

Section S5 MD SIMULATIONS

Force field parameters for the fluorescent probe were computed on the DFT(B3LYP-D3/def2-SVP) optimized structure of **1a** and **1b** using the program Antechamber implemented in AmberTools19.[31] AMBER atom types and RESP charges were used. The receptor was described via the AMBER ff14SB force field[33] and the lipids via the Lipid17 force field.[34] TIP3P water[25] parameters and the ion parameters of Li/Merz were used.[35] Periodic boundary conditions were employed with a cutoff of 10 Å for non-bonded interactions and the Particle Mesh Ewald method for long-range electrostatics. All the MD simulations were run using the AMBER18 software package.[31]

The ground state optimized probe forms were neutralized with one or two Na^+ ions for the **1b** and **1a** form, respectively, and solvated in a box of TIP3P water[25] with a minimum distance of 40 Å from the solute to the boundaries of the box using tLEaP.[31] Then, the system was minimized in three stages: protons, then solvent molecules and counterions, and finally, the whole system. Harmonic restraints of 200 kcal mol^{-1} $Å^{-2}$ were imposed to the rest of the atoms in each stage. After that, the system was thermally equilibrated for 20 ps from 100 K to 300 K using the Berendsen thermostat and restraining the solute with 20 kcal mol^{-1} $Å^{-2}$. Finally, and before production, the system was equilibrated in 6 steps from NVT to NPT ensemble for 38 ps. The system was further simulated for 100 ns with the Langevin thermostat with a time step of 0.2 fs. The simulation of the receptor in the membrane was executed following a similar protocol as for the probe in water. The protocol only deviates in the heating and equilibration process: The system was heated 200 ps from 0 K to 300 K using the Langevin thermostat with harmonic restraint imposed on all the atoms of receptor, probes, and lipids (10 kcal mol^{-1} $Å^{-2}$); the equilibration was executed in 10 steps for 5 ns in total. Finally, the system was further simulated for 100 ns.

Several parameters of the MD simulation, apart from the ones pictured in the main work, were analyzed. Figure S3 shows the distances d_1-d_3 along the 100 ns simulation. The choice of the initial receptor structure (PDB id. 6HUK vs. 6HUO) was validated in Figure S4. Figure S5 pictures snapshots of **1a** and **1b** at the GABA binding site B of the $GABA_AR$(α1/β3/γ2) and Figure S6 displays the root-means square deviation along the 100 ns simulation.

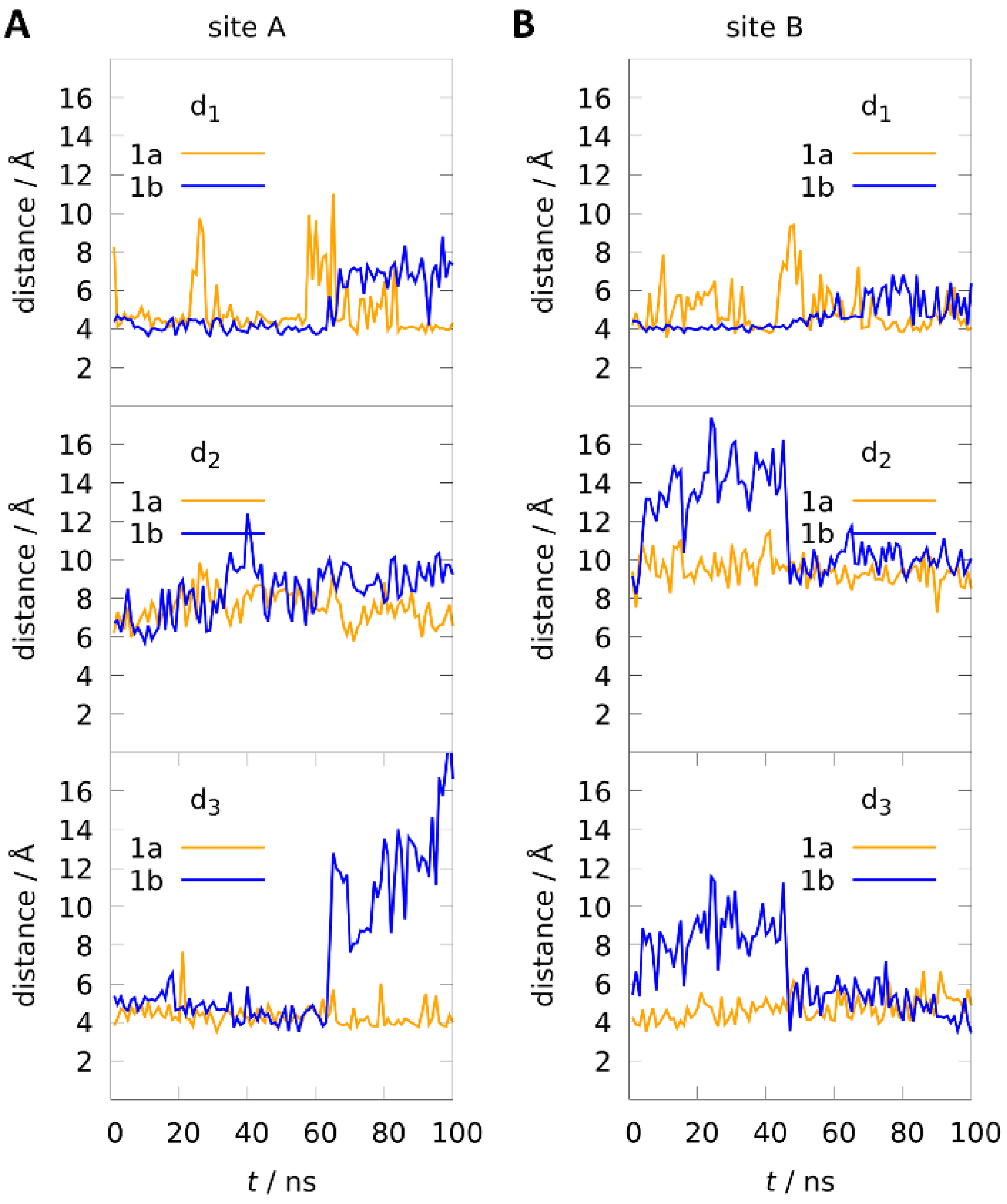

Figure S3. Plot of distances d_1-d_3 (Å) along the MD simulation of 100 ns for **1a** (orange) and **1b** (blue) in site A (A) and in site B (B).

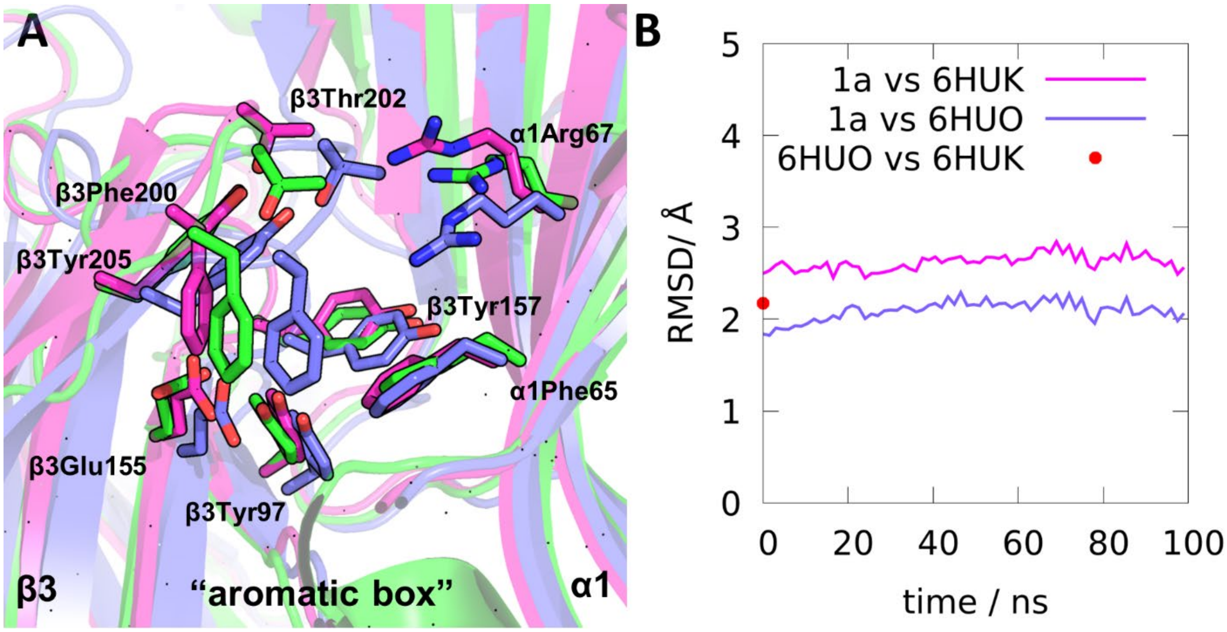

Figure S4. (A) Overlayed structures of the aromatic box at the GABA binding site A for PDB id. 6HUK[16] (magenta), PDB id. 6HUO[16] (purple), and one snapshot from the MD simulation of **1a** (green) (B) Plot of the root-means square deviation (RMSD, Å) of the backbone carbon atoms of the receptor along the MD simulation of **1a** of 100 ns against PDB id. 6HUK (magenta line) and PDB id. 6HUO (purple line). The RMSD of 6HUK against 6HUO is shown as a red dot.

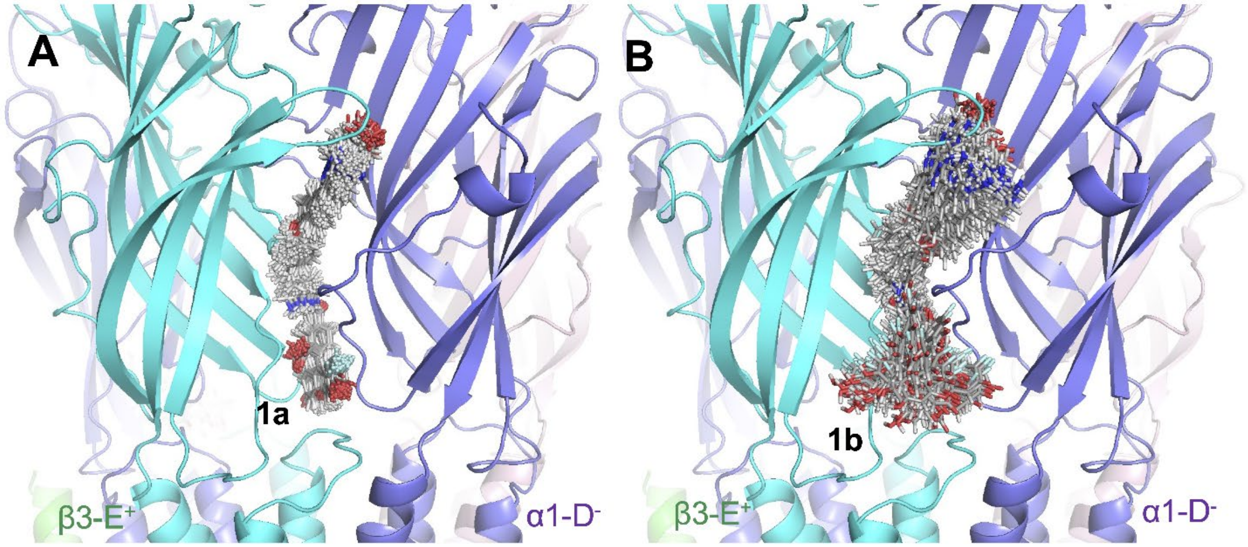

Figure S5. (A-B) Overlaid snapshots of **1a** (A) and **1b** (B) at the GABA binding site B of the $GABA_AR$(α1/β3/γ2) along the MD simulation of 100 ns.

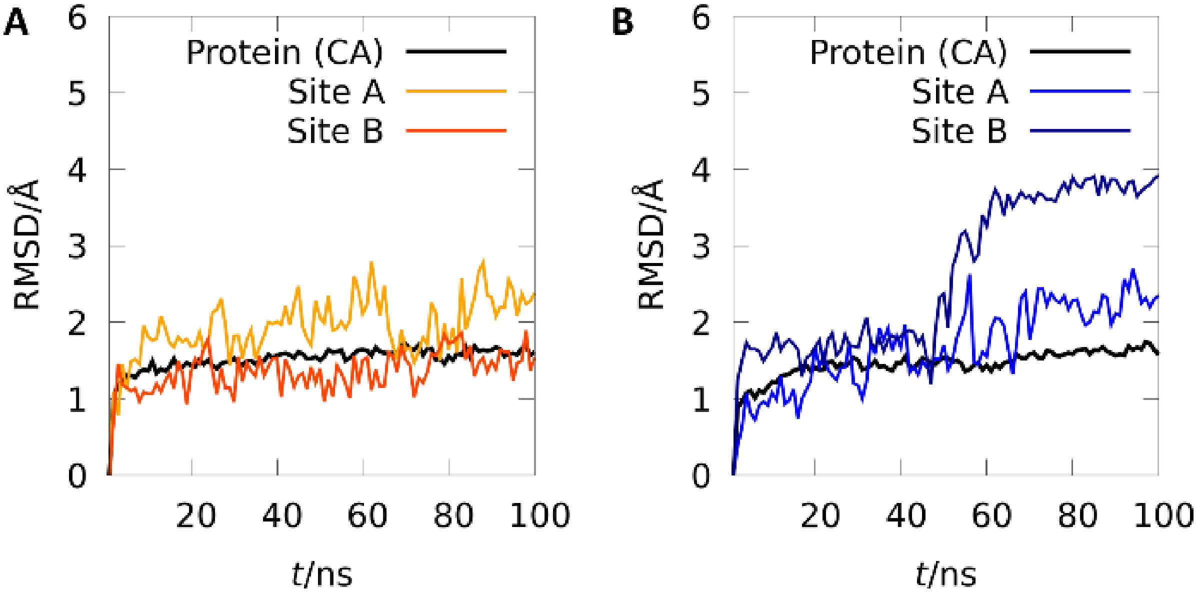

Figure S6. Plot of the root-means square deviation (RMSD, Å) of the backbone carbon atoms of the receptor (black line) along the MD simulation of 100 ns, as well as RMSD plots for **1a** (A) and **1b** (B) in site A and in site B for all atoms except hydrogen.

Section S6 BINDING ENERGY ANALYSIS

The binding free energies of **1a** and **1b** in binding sites A and B were calculated using the MM/ISMSA program.[36] It estimates the solvent-corrected binding energies (Table S4) as well as their per-residue decomposition (Table S5). The values reported in the results are averages. The last 15 ns of the MD simulations were considered for the calculation of the binding energies in accordance with their root-means square deviation (RMSD) plots (Figure S6).

**Table S4**. Computed binding energy (kcal mol^{-1}) for **1a** and **1b** at the GABA sites A and B.*					
**1a**					
site A					
**Van der Waals**	**Coulombic**	**Desolvation (Ligand)**	**Desolvation (Receptor)**	**Apolar**	**Total**
-75.82	-11.01	10.91	14.92	6.19	-67.18
site B					
**Van der Waals**	**Coulombic**	**Desolvation (Ligand)**	**Desolvation (Receptor)**	**Apolar**	**Total**
-56.03	-7.98	8.63	4.71	-4.45	-55.14
**1b**					
site A					
**Van der Waals**	**Coulombic**	**Desolvation (Ligand)**	**Desolvation (Receptor)**	**Apolar**	**Total**
-32.91	-3.36	6.54	6.84	-2.61	-25.50
site B					
**Van der Waals**	**Coulombic**	**Desolvation (Ligand)**	**Desolvation (Receptor)**	**Apolar**	**Total**
-43.67	-1.49	7.26	8.51	-3.27	-32.67

**Table S5**. Computed binding energy (kcal mol^{-1}) decomposed per residue for **1a** and **1b** at the GABA sites A and B.*			
**1a**			
**site A**		**site B**	
**Residue**	**Energy** (kcal mol^{-1})	**Residue**	**Energy** (kcal mol^{-1})
α1Arg187	-8.13 ± 2.58	αArg187	-5.53 ± 3.75
β3Met137	-4.23 ± 1.72	αAsn189	-4.36 ± 3.02
α1Arg67	-4.08 ± 1.35	αPhe46	-3.54 ± 2.48
α1Phe46	-3.61 ± 1.05	βLys102	-2.99 ± 2.30
β3Phe200	-3.37 ± 1.52	βMet137	-2.98 ± 2.41
**1b**			
**site A**		**site B**	
**Residue**	**Energy** (kcal mol^{-1})	**Residue**	**Energy** (kcal mol^{-1})
αArg187	-5.10 ± 0.86	βMet137	-4.62 ± 0.46
αPhe46	-4.59 ± 0.71	αPhe200	-3.62 ± 0.97
αAsp184	-2.82 ± 1.06	βMet138	-3.30 ± 0.39
αThr48	-2.26 ± 0.44	βMet55	-2.23 ± 0.28
αPhe65	-2.23 ± 0.53	βAsp139	-2.23 ± 0.41
*Energies computed using the last 15 ns of the MD simulations. The initial structures were not relaxed before the calculation of the binding energies.			

Section S7 QM/MM MD SIMULATIONS

As QM region, the OG488 part of **1a** (41 atoms) was treated using the third order semiempirical density-functional tight binding method DFTB3[41–43] implemented in AMBER18.[44] The environment (solvent, protein, and lipid membrane) was treated as point charges in the calculation of the energy of the QM part. First, taking the 100 ns MD simulations of **1a** bound to $GABA_AR$ and in a box of water molecules, one snapshot per system was randomly selected from the major conformational clusters (58% in site A in the membrane context and 58% in aqueous solution). The cluster analysis was performed using the *k*-means algorithm implemented in *cpptraj* for 3 clusters. Second, QM/MM MD simulations were run for 20 ps in the ground state (electronic state S_0) for both systems (see Scheme S1). SHAKE and Ewald were deactivated in the QM region and a cut-off of 15 Å (water)/30 Å ($GABA_AR$) was applied from the center of the QM region for the electrostatic term. Third, from the former trajectories on the ground state, 11 snapshots from the last 10 ps were taken and used as input geometries for a 500 fs Born-Oppenheimer MD simulation on the S_1 excited state (11 simulations for each system). The QM region, the OG488 part of **1a**, was treated with TD-DFT (TD-BP86-D3/def2-SVP)[45] using the interface AMBER18:ORCA.[46,47] The SlowConv algorithm and the deactivation of the SOSCF algorithm was required to get SCF convergences. 8 roots were included in the calculation and the energy of the potential energy surface (PES) of the S_1 state was used for the propagation. We also tested the use of B3LYP as DFT-functional for the QM/MM MD propagation on the S_1 PES. See Table S6 for the comparison. Although we obtain higher energies with B3LYP compared to BP86, we obtained similar results for oscillator strength (f_{osc}), charge transfer numbers (CT), participation ratio (PR) numbers for the initial/final electron delocalization, and transition character (not shown). We conclude that the geometry of the molecule is well enough described by BP86.

**Table S6**. First excited electronic state (S_1) properties of 5 snapshots obtained on the S_1 PES using BP86 and B3LYP as DFT functional in the trajectory of **1a** at the $GABA_AR$.* The emission energies were calculated with B3LYP as described in the text.					
**BP86**					
**Snapshot**	**ΔE_{Em}** [eV]	**f_{osc}** [arb.u.]	**PR_i**	**PR_f**	**CT**
1	1.77	0.03	1.26	1.12	0.88
2	1.64	0.01	1.24	1.13	0.89
3	1.59	0.01	1.20	1.12	0.91
4	1.71	0.03	1.25	1.10	0.88
5	1.76	0.03	1.21	1.09	0.90
**B3LYP**					
**Snapshot**	**ΔE_{Em}** [eV]	**f_{osc}** [arb.u.]	**PR_i**	**PR_f**	**CT**
1	1.95	0.07	1.39	1.11	0.83
2	2.19	0.04	1.27	1.10	0.88
3	2.05	0.04	1.26	1.09	0.88
4	2.12	0.07	1.33	1.10	0.85
5	2.06	0.09	1.43	1.11	0.82
*Only the fluorophore is in the QM region. The snapshots were randomly selected.					

The emission spectrum for **1a** was calculated using 5 snapshots from the last 250 fs of each of the 11 S_1 excited state Born-Oppenheimer MD simulations in aqueous solution and the protein context (55 snapshots each, see Scheme S1, see repository for xyz-structures: https://phaidra.univie.ac.at/o:1383031). Each snapshot was then used for a TD-DFT single point calculation using Gaussian 16, v. A.01[5] (B3LYP-D3/def2-SVP) and an electrostatic cut-off of 55 Å (see Section S1 for the discussion about the DFT-functional selection). The QM region is composed of the whole probe in aqueous solution and of only the fluorophore in the protein context. The BP86 functional is sufficient for an efficient S_1 propagation, but not for the calculation of the emission spectrum, as it delivers drastic red-shifts. The emission energy is calculated as the vertical excitation energy from ground to first excited state in the optimized S_1 geometry. The oscillator strength is used as an approximation for the emission intensity.[48–50] The spectra were convoluted by Lorentzian functions weighted by the oscillator strength with a full width at half maximum of 0.10 eV using TheoDORE[51] (see Figure 5A). To one more time validate the use of the B3LYP functional, a second spectrum of **1a** was convoluted based on M06-D3/def2-SVP calculations (30 snapshots). The comparison between B3LYP and M06 is shown in Figure S7. The spectrum with the M06 functional is shifted to higher energies compared to B3LYP, but the characteristics of the spectrum stay consistent.

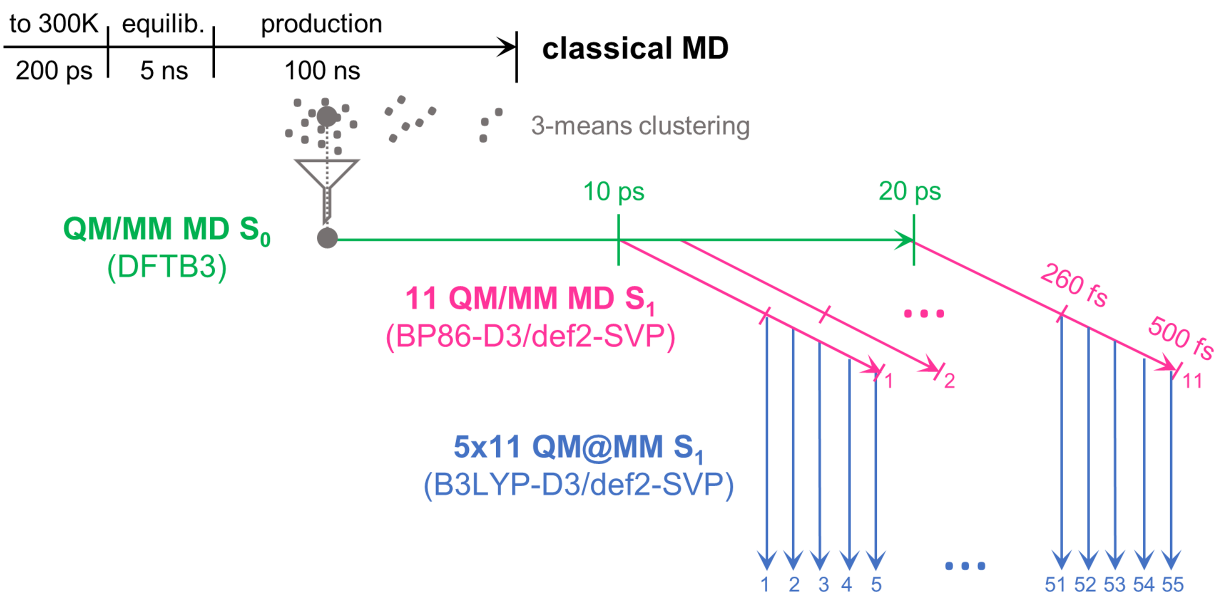

Scheme S1. Schematic workflow of how the 55 snapshots for the emission spectrum of the probe at the receptor were obtained using classical MD, QM/MM MD and QM@MM methods.

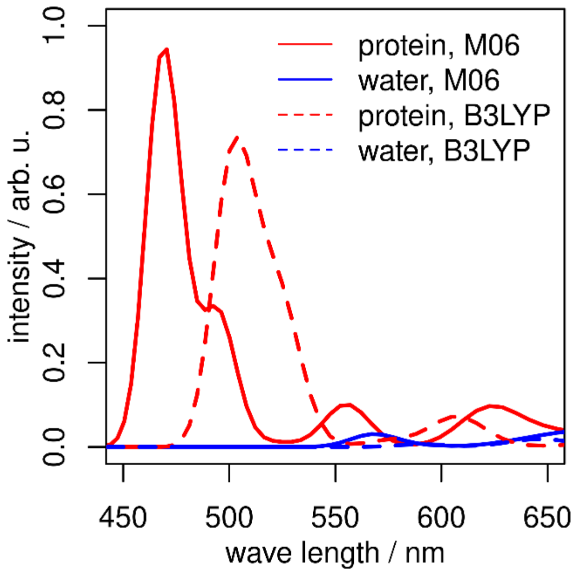

Figure S7. Computed emission spectra of **1a** bound to the $GABA_AR$ (red lines) and in water solution (blue lines) at the TD-M06-D3/def2-SVP level of theory (solid lines) compared to the TD-B3LYP-D3/def2-SVP level of theory (dashed lines).

BIBLIOGRAPHY

[1] A. D. Becke, *Phys. Rev. A* **1988**, *38*, 3098–3100.
[2] C. Lee, W. Yang, R. G. Parr, *Phys. Rev. B* **1988**, *37*, 785–789.
[3] Y. Zhao, D. G. Truhlar, *Theor. Chem. Acc.* **2008**, *120*, 215–241.
[4] T. Yanai, D. P. Tew, N. C. Handy, *Chem. Phys. Lett.* **2004**, *393*, 51–57.
[5] M. J. Frisch, G. W. Trucks, H. B. Schlegel, G. E. Scuseria, M. A. Robb, J. R. Cheeseman, G. Scalmani, V. Barone, G. A. Petersson, H. Nakatsuji, X. Li, M. Caricato, A. V Marenich, J. Bloino, B. G. Janesko, R. Gomperts, B. Mennucci, H. P. Hratchian, J. V Ortiz, A. F. Izmaylov, J. L. Sonnenberg, D. Williams-Young, F. Ding, F. Lipparini, F. Egidi, J. Goings, B. Peng, A. Petrone, T. Henderson, D. Ranasinghe, V. G. Zakrzewski, J. Gao, N. Rega, G. Zheng, W. Liang, M. Hada, M. Ehara, K. Toyota, R. Fukuda, J. Hasegawa, M. Ishida, T. Nakajima, Y. Honda, O. Kitao, H. Nakai, T. Vreven, K. Throssell, J. A. Montgomery Jr., J. E. Peralta, F. Ogliaro, M. J. Bearpark, J. J. Heyd, E. N. Brothers, K. N. Kudin, V. N. Staroverov, T. A. Keith, R. Kobayashi, J. Normand, K. Raghavachari, A. P. Rendell, J. C. Burant, S. S. Iyengar, J. Tomasi, M. Cossi, J. M. Millam, M. Klene, C. Adamo, R. Cammi, J. W. Ochterski, R. L. Martin, K. Morokuma, O. Farkas, J. B. Foresman, D. J. Fox, *Gaussian16 Revis. A.03, Gaussian Inc., Wallingford CT,* **2016**.
[6] A. Schäfer, H. Horn, R. Ahlrichs, *J. Chem. Phys.* **1992**, *97*, 2571–2577.
[7] S. Grimme, J. Antony, S. Ehrlich, H. Krieg, *J. Chem. Phys.* **2010**, *132*, 154104.
[8] S. Miertuš, E. Scrocco, J. Tomasi, *Chem. Phys.* **1981**, *55*, 117–129.
[9] S. Miertuš, J. Tomasi, *Chem. Phys.* **1982**, *65*, 239–245.
[10] J. L. Pascual-ahuir, E. Silla, I. Tuñon, *J. Comput. Chem.* **1994**, *15*, 1127–1138.
[11] M. Caricato, B. Mennucci, J. Tomasi, F. Ingrosso, R. Cammi, S. Corni, G. Scalmani, *J. Chem. Phys.* **2006**, *124*, 124520.
[12] University of Karlsruhe and Forschungszentrum Karlsruhe GmbH 1989-2007, TURBOMOLE GmbH since 2007, "TURBOMOLE V7.0 2015," can be found under http://www.turbomole.com, **2015**.
[13] A. Klamt, C. Moya, J. Palomar, *J. Chem. Theory Comput.* **2015**, *11*, 4220–4225.
[14] S. Sakamoto, K. Yamaura, T. Numata, F. Harada, K. Amaike, R. Inoue, S. Kiyonaka, I. Hamachi, *ACS Cent. Sci.* **2019**, *5*, 1541–1553.
[15] A. D. Becke, *J. Chem. Phys.* **1993**, *98*, 5648–5652.
[16] S. Masiulis, R. Desai, T. Uchański, I. Serna Martin, D. Laverty, D. Karia, T. Malinauskas, J. Zivanov, E. Pardon, A. Kotecha, J. Steyaert, K. W. Miller, A. R. Aricescu, *Nature* **2019**, *565*, 454–459.
[17] P. S. Miller, S. Masiulis, T. Malinauskas, A. Kotecha, S. Rao, S. Chavali, L. De Colibus, E. Pardon, S. Hannan, S. Scott, Z. Sun, B. Frenz, G. Klesse, S. Li, J. M. Diprose, C. A. Siebert, R. M. Esnouf, F. DiMaio, S. J. Tucker, T. G. Smart, J. Steyaert, M. M. Babu, M. S. P. Sansom, J. T. Huiskonen, A. R. Aricescu, *bioRxiv* **2018**, 338343.
[18] M. Jansen, M. Bali, M. H. Akabas, *J. Gen. Physiol.* **2008**, *131*, 137–146.
[19] P. S. Miller, A. R. Aricescu, *Nature* **2014**, *512*, 270–275.
[20] A. Onufriev, B. Aguilar, R. Anandakrishnan, **2019**, H++ Server, Version~3.2.
[21] J. C. Gordon, J. B. Myers, T. Folta, V. Shoja, L. S. Heath, A. Onufriev, *Nucleic Acids Res.* **2005**, *33*, W368–W371.
[22] J. Myers, G. Grothaus, S. Narayanan, A. Onufriev, *Proteins Struct. Funct. Bioinforma.* **2006**, *63*, 928–938.
[23] R. Anandakrishnan, B. Aguilar, A. V Onufriev, *Nucleic Acids Res.* **2012**, *40*, W537–W541.
[24] M. A. Lomize, I. D. Pogozheva, H. Joo, H. I. Mosberg, A. L. Lomize, *Nucleic Acids Res.* **2012**, *40*, D370–D376.
[25] W. L. Jorgensen, J. Chandrasekhar, J. D. Madura, R. W. Impey, M. L. Klein, *J. Chem. Phys.* **1983**, *79*, 926–935.
[26] S. Jo, T. Kim, W. Im, *PLoS One* **2007**, *2*, e880.

[27] S. Jo, T. Kim, V. G. Iyer, W. Im, *J. Comput. Chem.* **2008**, *29*, 1859–1865.
[28] S. Jo, J. B. Lim, J. B. Klauda, W. Im, *Biophys. J.* **2009**, *97*, 50–58.
[29] E. L. Wu, X. Cheng, S. Jo, H. Rui, K. C. Song, E. M. Dávila-Contreras, Y. Qi, J. Lee, V. Monje-Galvan, R. M. Venable, J. B. Klauda, W. Im, *J. Comput. Chem.* **2014**, *35*, 1997–2004.
[30] J. Lee, D. S. Patel, J. Ståhle, S.-J. Park, N. R. Kern, S. Kim, J. Lee, X. Cheng, M. A. Valvano, O. Holst, Y. A. Knirel, Y. Qi, S. Jo, J. B. Klauda, G. Widmalm, W. Im, *J. Chem. Theory Comput.* **2019**, *15*, 775–786.
[31] D. A. Case, I. Y. Ben-Shalom, S. R. Brozell, D. S. Cerutti, T. E. Cheatham, III, V. W. D. Cruzeiro, T. A. Darden, R. E. Duke, D. Ghoreishi, M. K. Gilson, H. Gohlke, A. W. Goetz, D. Greene, R. Harris, N. Homeyer, S. Izadi, A. Kovalenko, T. Kurtzman, T. S. Lee, S. LeGrand, P. Li, C. Lin, J. Liu, T. Luchko, R. Luo, D. J. Mermelstein, K. M. Merz, Y. Miao, G. Monard, C. Nguyen, H. Nguyen, I. Omelyan, A. Onufriev, F. Pan, R. Qi, D. R. Roe, A. Roitberg, C. Sagui, S. Schott-Verdugo, J. Shen, C. L. Simmerling, J. Smith, R. Salomon-Ferrer, J. Swails, R. C. Walker, J. Wang, H. Wei, R. M. Wolf, X. Wu, L. Xiao, D. M. York, P. A. Kollman, *AMBER18, Univ. California, San Fr. ,* **2018**.
[32] D. Laverty, R. Desai, T. Uchański, S. Masiulis, W. J. Stec, T. Malinauskas, J. Zivanov, E. Pardon, J. Steyaert, K. W. Miller, A. R. Aricescu, *Nature* **2019**, *565*, 516–520.
[33] J. A. Maier, C. Martinez, K. Kasavajhala, L. Wickstrom, K. E. Hauser, C. Simmerling, *J. Chem. Theory Comput.* **2015**, *11*, 3696–3713.
[34] C. J. Dickson, B. D. Madej, Å. A. Skjevik, R. M. Betz, K. Teigen, I. R. Gould, R. C. Walker, *J. Chem. Theory Comput.* **2014**, *10*, 865–879.
[35] P. Li, B. P. Roberts, D. K. Chakravorty, K. M. Merz, *J. Chem. Theory Comput.* **2013**, *9*, 2733–2748.
[36] J. Klett, A. Núñez-Salgado, H. G. Dos Santos, Á. Cortés-Cabrera, A. Perona, R. Gil-Redondo, D. Abia, F. Gago, A. Morreale, *J. Chem. Theory Comput.* **2012**, *8*, 3395–3408.
[37] C. Wang, Q. Qiao, W. Chi, J. Chen, W. Liu, D. Tan, S. McKechnie, D. Lyu, X.-F. Jiang, W. Zhou, N. Xu, Q. Zhang, Z. Xu, X. Liu, *Angew. Chemie Int. Ed.* **2020**, *59*, 10160–10172.
[38] M. A. Gerasimova, F. N. Tomilin, E. Y. Malyar, S. A. Varganov, D. G. Fedorov, S. G. Ovchinnikov, E. A. Slyusareva, *Dye. Pigment.* **2020**, *173*, 107851.
[39] D. Zúñiga-Núñez, R. A. Zamora, P. Barrias, C. Tirapegui, H. Poblete, G. Cárdenas-Jirón, E. I. Alarcon, A. Aspée, *Phys. Chem. Chem. Phys.* **2018**, *20*, 27621–27629.
[40] A. M. El-Zohry, E. A. Orabi, M. Karlsson, B. Zietz, *J. Phys. Chem. A* **2021**, *125*, 2885–2894.
[41] M. Gaus, Q. Cui, M. Elstner, *J. Chem. Theory Comput.* **2011**, *7*, 931–948.
[42] M. Gaus, A. Goez, M. Elstner, *J. Chem. Theory Comput.* **2013**, *9*, 338–354.
[43] M. Kubillus, T. Kubař, M. Gaus, J. Řezáč, M. Elstner, *J. Chem. Theory Comput.* **2015**, *11*, 332–342.
[44] A. W. Götz, M. A. Clark, R. C. Walker, *J. Comput. Chem.* **2014**, *35*, 95–108.
[45] F. Weigend, *Phys. Chem. Chem. Phys.* **2006**, *8*, 1057.
[46] F. Neese, *Wiley Interdiscip. Rev. Comput. Mol. Sci.* **2012**, *2*, 73–78.
[47] F. Neese, *WIREs Comput. Mol. Sci.* **2018**, *8:e1327*, DOI 10.1002/wcms.1327.
[48] G. Cardenas, M. F. S. J. Menger, N. Ramos-Berdullas, P. A. Sánchez-Murcia, *Chem. – A Eur. J.* **2021**, *27*, 4927–4931.
[49] J. Preiss, D. Kage, K. Hoffmann, T. J. Martínez, U. Resch-Genger, M. Presselt, *J. Phys. Chem. A* **2018**, *122*, 9813–9820.
[50] Z. C. Wong, W. Y. Fan, T. S. Chwee, M. B. Sullivan, *RSC Adv.* **2016**, *6*, 87237–87245.
[51] F. Plasser, *J. Chem. Phys.* **2020**, *152*, 084108.
